# Barriers and facilitators to non-pharmacological management of metabolic dysfunction-associated steatotic liver disease: a qualitative evidence synthesis

**DOI:** 10.3389/fphar.2025.1615809

**Published:** 2025-08-29

**Authors:** Yusuf Yilmaz

**Affiliations:** ^1^ Department of Gastroenterology, School of Medicine, Recep Tayyip Erdoğan University, Rize, Türkiye; ^2^ The Global NASH Council, Washington, DC, United States

**Keywords:** metabolic dysfunction-associated steatotic liver disease, lifestyle interventions, barriers, facilitators, qualitative evidence synthesis

## Abstract

Metabolic dysfunction-associated steatotic liver disease (MASLD) represents a significant global health concern, with limited pharmacological options despite extensive research efforts. While the recent conditional approval of resmetirom for metabolic dysfunction-associated steatohepatitis with significant or advanced fibrosis has marked a major therapeutic milestone, lifestyle interventions–primarily dietary modifications and structured physical activity–remain the foundation of MASLD management for most patients. However, integrating these non-pharmacological strategies into routine clinical practice remains a significant challenge. In this qualitative evidence synthesis, we searched the PubMed, Scopus, ScienceDirect, and Google Scholar databases to identify and categorize the principal barriers and facilitators influencing the implementation of lifestyle interventions in MASLD care. The analysis identified 67 barriers and 64 facilitators. To address these multifaceted challenges, we propose a multidisciplinary management framework anchored in six core principles: (1) strategic integration of diverse professional expertise with clear role delineation; (2) patient-centered interventions that address both societal and individual barriers while leveraging facilitators; (3) early preventive measures to halt disease progression prior to the development of significant fibrosis; (4) tailored approaches responsive to disease severity and comorbidities; (5) optimized monitoring protocols with specific thresholds for intervention adjustment; and (6) judicious incorporation of digital health technologies, accounting for variability in digital literacy. We conclude that understanding both barriers and facilitators is essential for developing adaptable, patient-centered interventions. Our findings may provide a roadmap for addressing implementation challenges in non-pharmacological MASLD management, emphasizing the importance of preventive, tailored, multidisciplinary approaches that begin early and evolve with disease progression.

## 1 Introduction

Metabolic dysfunction-associated steatotic liver disease (MASLD) is currently recognized as the most common chronic liver disease worldwide, affecting an estimated 38% of the adult population (Stefan et al., 2025; Younossi et al., 2023). This substantial burden has positioned MASLD as a major public health challenge and a leading indication for liver transplantation (Gadiparthi et al., 2020). According to recent data from 204 countries, year 2021 witnessed approximately 1,267.9 million MASLD cases worldwide, resulting in 138.3 thousand deaths and 3,667.3 thousand disability-adjusted life years (DALYs) (Huang et al., 2025). Concurrently, the global age-standardized prevalence reached 15,018.1 per 100,000 population, while mortality and DALYs rates were recorded at 1.6 and 42.4 per 100,000 population, respectively (Huang et al., 2025). These metrics represent significant increases of 24.3% in prevalence and 5.5% in both mortality and DALY rates since 1990, underscoring the accelerating impact of this condition on global health outcomes (Huang et al., 2025). Importantly, this epidemiological trend translates into considerable economic strain, with annual direct medical costs in the United States alone estimated to exceed $100 billion (Miao et al., 2024).

Despite substantial progress in understanding the pathophysiology of MASLD, pharmacological treatment options have remained limited (Puengel and Tacke, 2024). A significant breakthrough has been recently achieved with the conditional FDA approval of resmetirom, a selective thyroid hormone receptor-beta agonist (Keam, 2024). This novel agent induces localized hyperthyroid effects in the liver, effectively reducing lipid accumulation and fibrosis in patients with metabolic dysfunction-associated steatohepatitis (MASH) who have significant or advanced fibrosis (Kaya et al., 2025; Au et al., 2024). However, the clinical use of resmetirom is hindered by several challenges–including restricted distribution to specialty pharmacies, high retail costs, and complex preauthorization requirements from payors, thereby complicating the prescribing process and involving multiple stakeholders (Ravela et al., 2025). In this scenario, non-pharmacological interventions–particularly weight reduction through controlled caloric intake and physical activity–continue to represent the foundation of MASLD management for most affected patients (Rajewski et al., 2025; Ali et al., 2024; Stefano et al., 2023; Ratziu, 2017). Current evidence supports targeted weight reduction of 7%–10% of total body weight for most patients, with the 10% threshold specifically recommended for those with at-risk MASH, as this degree of weight loss consistently correlates with significant improvements in hepatic histology (Diaz et al., 2025). For patients with lean MASLD phenotype, a more modest weight reduction of 3%–5% may also confer measurable hepatic benefits (Diaz et al., 2025). Nutritional interventions with established efficacy include the Mediterranean dietary pattern and carbohydrate-restricted approaches–which emphasize limitation of refined carbohydrates and added sugars, particularly fructose, which has been implicated in hepatic *de novo* lipogenesis (Simancas-Racines et al., 2025). Specifically, clinical recommendations include minimizing consumption of sugar-sweetened beverages, ultra-processed foods, and products containing high-fructose corn syrup (Parra-Vargas et al., 2020). Notably, the therapeutic efficacy of weight loss can be optimized through the synergistic implementation of both dietary modification and structured physical activity regimens (Chen et al., 2025). Both aerobic exercise modalities (e.g., walking, jogging, cycling, swimming) (Mambrini et al., 2024; Machado, 2021) and resistance training protocols (Medeiros et al., 2025; Askari et al., 2025) have demonstrated efficacy in improving hepatic steatosis and metabolic parameters in patients with MASLD. For adults without cardiovascular or musculoskeletal contraindications, current evidence supports 150–300 min weekly of moderate-intensity aerobic activity, 75–150 min weekly of vigorous-intensity aerobic activity, or an equivalent combination thereof (Diaz et al., 2025). Concurrent resistance training performed ≥2 days weekly can also provide complementary metabolic benefits that improve hepatic and systemic outcomes (Diaz et al., 2025).

Despite robust evidence supporting non-pharmacological interventions for MASLD, their implementation in contemporary clinical settings remains suboptimal due to multiple complex challenges at both societal and individual levels. These hurdles may include–but are not limited to–organizational constraints, limited healthcare provider training, patient adherence difficulties, and insufficient integration into routine clinical workflows (Deshpande et al., 2024; Shibayama et al., 2024; Gu et al., 2023; Heredia et al., 2022; Arora et al., 2021; Stine et al., 2021; Haigh et al., 2019). Building on these premises, we carried out a qualitative evidence synthesis with three primary objectives: 1) to investigate and analyze the societal and individual barriers that may impede the implementation of non-pharmacological interventions for MASLD; 2) to characterize the facilitators that can enhance intervention adherence and effectiveness; and 3) to develop a multidisciplinary framework for integrating lifestyle interventions into MASLD clinical care pathways. Our overarching goals were to inform clinical practice standards, to guide healthcare policy development, and to stimulate targeted research initiatives that may advance the implementation of non-pharmacological management strategies across the MASLD spectrum.

## 2 Methods

A qualitative evidence synthesis was conducted through searches of PubMed, Scopus, ScienceDirect, and Google Scholar electronic databases. The literature review encompassed original research articles, reviews, meta-analyses, and systematic reviews published in English-language journals between 1 January 2015, and 15 April 2025. Selection criteria specifically targeted non-pharmacological interventions for the prevention and management of MASLD, with emphasis on dietary modification strategies and physical activity interventions. Excluded were conference abstracts, case reports, editorials, letters, and non-peer-reviewed materials (e.g., preprints). The detailed search strategy and keywords are presented in Table 1, while Table 2 describes the construction of search strings and the application of filters for each database. Following an initial screening of titles and abstracts, full-text articles deemed potentially relevant were assessed. Data extraction prioritized identifying barriers and facilitators influencing the implementation of non-pharmacological interventions. A thematic synthesis approach was used to analyze the extracted data on barriers and facilitators. Relevant excerpts from the included studies were inductively coded to identify recurring patterns and concepts related to the non-pharmacological management of MASLD. Codes were compared across studies and refined through an iterative process to ensure consistency and rigor in theme development. Thematic analysis continued until no new themes emerged, indicating thematic saturation. The identified barriers and facilitators were then systematically reviewed and organized using a dual-level taxonomy that distinguishes between individual-level and societal-level determinants. Finally, the findings were synthesized narratively to inform the design of a multidisciplinary framework for integrating lifestyle interventions into MASLD clinical care pathways.

**TABLE 1:** Search strategy and keywords.

Concept category	Search terms
Disease terminology	“MASLD” OR “MAFLD” OR “NAFLD” OR “Non-alcoholic fatty liver disease” OR “Metabolic-dysfunction associated fatty liver disease” OR “Metabolic dysfunction-associated steatotic liver disease” OR “fatty liver” OR “hepatic steatosis” OR “steatohepatitis” OR “NASH” OR “MASH”
Non-pharmacological interventions	“non-pharmacological” OR “lifestyle intervention*” OR “lifestyle modification*” OR “lifestyle therapy” OR “behavior* change” OR “self-management”
Dietary interventions	“diet*” OR “nutrition*” OR “Mediterranean diet” OR “caloric restriction” OR “intermittent fasting” OR “ketogenic” OR “low carbohydrate” OR “dietary pattern*” OR “macronutrient*” OR “micronutrient*”
Physical activity interventions	“exercise” OR “physical activity” OR “aerobic training” OR “resistance training” OR “fitness” OR “sedentary behavior” OR “cardiorespiratory fitness”
Barriers and facilitators	“barrier*” OR “facilitator*” OR “adherence” OR “compliance” OR “implementation” OR “effectiveness” OR “challenge*” OR “obstacle*” OR “enabler*”
Clinical management	“management” OR “care pathway*” OR “clinical pathway*” OR “framework” OR “guideline*” OR “health policy” OR “multidisciplinary” OR “integrated care” OR “patient education”

**TABLE 2:** Search strings.

Database	Search strings	Filters
PubMed	(Disease terminology) AND (Non-pharmacological interventions OR Dietary interventions OR Physical activity interventions) AND (Barriers and facilitators OR Clinical management)	Date: 1 January 2015 - 15 April 2025; Article types: Original research, Meta-analyses, Reviews, Systematic reviews; Language: English
Scopus	TITLE-ABS-KEY ((Disease terminology) AND (Non-pharmacological interventions OR Dietary interventions OR Physical activity interventions) AND (Barriers and facilitators OR Clinical management))	Date: 2015–2025; Document type: Article, Review; Language: English
ScienceDirect	(Selected terms from Disease terminology) AND (Selected terms from interventions) AND (Selected terms from barriers/facilitators and management)	Years: 2015–2025; Types: Research articles, Review articles
Google Scholar	(Selected terms from Disease terminology) AND (Selected terms from interventions) AND (Selected terms from barriers/facilitators and management)	Custom range: 2015–2025

## 3 Barriers and facilitators to implementing exercise and nutritional strategies in patients with MASLD

The analysis identified 67 barriers (Table 3) and 64 facilitators (Table 4) relevant to the implementation of non-pharmacological interventions for MASLD. For descriptive clarity, these factors were classified as either societal or individual. Importantly, the relationship between societal and individual factors was characterized by substantial interconnectedness, with numerous elements demonstrating bidirectional influences. In this context, addressing individual-level barriers may contribute to alleviating broader societal challenges, while interventions at the societal level have the potential to diminish individual obstacles. For instance, healthcare system barriers such as “limited reimbursement for lifestyle interventions” (Gobble et al., 2022; Freeman et al., 2021) at the societal level directly impact individual-level treatment accessibility, while psychological factors like “low motivation for sustained lifestyle change” (Tincopa et al., 2021; Hardcastle et al., 2015) at the individual level can undermine the effectiveness of even well-designed societal interventions. Notably, the implementation of non-pharmacological interventions for MASLD revealed a complex interplay where factors frequently function as both barriers and facilitators depending on context, implementation strategy, and specific circumstances. This duality creates a dynamic intervention landscape requiring nuanced understanding for effective clinical practice. The digital environment presents a notable example of this dual role phenomenon. In this regard, digital technologies appear in both tables–as potential barriers when characterized by “limited access to evidence-based digital health tools” (Giebel et al., 2023) and “poor integration of lifestyle tracking apps with healthcare systems” (Magrabi et al., 2019), yet simultaneously as facilitators through “mobile apps for tracking dietary intake and physical activity” (Kamel Boulos and Yang, 2021; Coughlin et al., 2015) and “telehealth platforms connecting patients with multidisciplinary teams” (Belber et al., 2023). This duality highlights how technological solutions can either hinder or enhance MASLD management depending on implementation, accessibility, and digital literacy levels. Cultural factors similarly operate at this intersection, functioning as barriers when “cultural dietary patterns high in refined carbohydrates and saturated fats” (Zhang et al., 2024; Clemente-Suárez et al., 2023) and “social gatherings centered around unhealthy eating” (Buksh et al., 2022; Levy et al., 2021) prevail. However, when cultural elements are integrated into intervention design through “cultural adaptation of lifestyle interventions” (Sevilla-González et al., 2025; Siddiqui et al., 2018) and “partnerships with community organizations,” (Jones et al., 2023; Lazarus et al., 2023) they become powerful facilitators for sustainable behavior change. Health problems themselves demonstrate this complex relationship. While conditions like “fatigue and reduced exercise capacity associated with MASLD” (Younossi et al., 2024) and “comorbidities limiting exercise options” (Deshpande et al., 2024) represent significant barriers, they simultaneously create opportunities for targeted interventions when addressed through “personalized exercise prescriptions based on fitness level and liver health” (Akuta et al., 2024; Glass et al., 2022) and “adapted exercise protocols for patients with comorbidities” (Farrugia et al., 2023). With respect to intervention design and implementation, physical activity programs for MASLD require well-designed approaches that acknowledge both barriers and facilitators. Within this framework, effective exercise programs must transcend the universal barriers of “increasing sedentary lifestyle” (Kim et al., 2020; Hallsworth and Adams, 2019) and “limited time for physical activity” (Patel et al., 2024) through tailored solutions that include “combined aerobic and resistance training programs” (Hashida et al., 2017) and “strategies for integrating physical activity into daily routines” (Arlinghaus and Johnston, 2018). Conversely, the emphasis on “supervised exercise sessions with progressive intensity increases” (Keating et al., 2023) reflects the need to address psychological barriers including “low motivation for sustained lifestyle change” (Livia et al., 2016) and “poor self-efficacy for exercise” (Frith et al., 2010). Nutritional interventions demonstrate similar complexity. Against barriers like “food industry influence promoting ultra-processed foods” (Grinshpan et al., 2023) and “disparities in access to healthy foods” (Kardashian et al., 2022), effective facilitators include adoption of the “Mediterranean dietary pattern emphasizing plant foods and healthy fats” (Zelber-Sagi et al., 2017) and “evidence-based nutritional advice” (Hydes et al., 2020). These approaches must be complemented by “techniques for navigating social eating situations” (Buksh et al., 2022) to overcome the barrier of “social gatherings centered around unhealthy eating” (Levy et al., 2021). The healthcare system itself encompasses significant barriers, including the “paucity of specialized MASLD clinics offering multidisciplinary care” (McPherson et al., 2022). These systemic challenges are compounded by healthcare professional practice barriers such as “inadequate training of physicians in nutrition science” (Mogre et al., 2018) and “focus on pharmacological rather than non-pharmacological interventions” (Hossain et al., 2016). Addressing these challenges requires facilitators that include “implementation of multidisciplinary MASLD care models” (Zoncapè et al., 2022) and “expanded reimbursement for evidence-based lifestyle interventions” (Patel, 2025). Professional education facilitators like “enhanced nutrition and exercise science education in medical curricula” (Khiri and Howells, 2025; Mealy et al., 2019) and “training in motivational interviewing and health behavior change techniques” (Bischof et al., 2021) directly target the identified barriers in clinical practice. Our results also revealed that current digital technologies present paradoxical implications in MASLD management, simultaneously offering therapeutic potential while introducing implementation challenges and possible adverse consequences. Accordingly, while barriers like “low digital health literacy” (Arias López et al., 2023) and “information overload from unverified online sources” (Figueroa et al., 2024) exist, thoughtfully designed technological facilitators can transform patient care through “wearable devices monitoring metabolic health parameters” (Keshet et al., 2023) and “virtual coaching for personalized exercise and nutrition guidance” (Buzcu et al., 2024; Albers et al., 2023) Behavioral support emerges as another critical domain addressing psychological barriers. Against challenges like “emotional eating patterns” (Ley et al., 2021) and “immediate reward preferences over long-term health benefits” (Michaelsen and Esch, 2023) effective facilitators include “motivational interviewing to address ambivalence about lifestyle change” (Almansour et al., 2023) and “cognitive behavioral strategies for managing emotional eating” (Smith et al., 2023). At the broader societal level, factors like “limited public health measures addressing metabolic health” (Díaz et al., 2022) and “socioeconomic barriers to gym memberships and exercise facilities” (Salmi et al., 2023) create substantial impediments to effective MASLD management. These challenges necessitate policy-level facilitators including “development of policies promoting healthy lifestyles” (Lobczowska et al., 2022) and “food policy initiatives targeting ultra-processed food reduction” (Popkin et al., 2021). Social inequalities represent particularly persistent barriers, with “higher prevalence of MASLD in marginalized populations with fewer resources” (Lorek et al., 2025) and “unequal access to healthcare professionals with MASLD expertise” (Miller et al., 2025). Addressing these disparities requires concerted efforts through facilitators like “community-based metabolic health education programs” (Okube et al., 2022) and “expanded funding for lifestyle intervention research” (Pagoto, 2011). The multidimensional nature of MASLD barriers and facilitators suggests that integrated approaches are essential for effective management. The implementation of “4P medicine (preventive, participatory, personalized, predictive)” (Lonardo et al., 2021) as a facilitator directly addresses multiple barriers across both societal and individual levels. Similarly, “personalized meal plans addressing individual metabolic profiles” (Marin-Alejandre et al., 2021) counters barriers related to conflicting nutritional messaging and cultural resistance to dietary modifications. The facilitator of “expanded preventive medicine approaches focusing on metabolic health” (Schattenberg et al., 2023) directly counteracts the barrier of “insufficient screening protocols for early MASLD detection” (Ilagan-Ying et al., 2023). Based on the available evidence, it is imperative to prioritize preventive approaches rather than focusing exclusively on treating patients with MASLD. However, expanding access to preventive care requires addressing fundamental societal barriers including “socioeconomic barriers” (Nasr et al., 2024) and “disparities in access to healthy foods” (Drisdelle et al., 2020). Importantly, the complexity of MASLD management necessitates coordinated efforts across multiple sectors and levels. While individual-level interventions like “self-monitoring tools for tracking progress” (Carpenter et al., 2022) are essential, they must be complemented by societal-level facilitators such as “public education campaigns on MASLD risk factors” (Balakrishnan and Rehm, 2024) and “workplace wellness initiatives addressing nutrition and physical activity” (Peñalvo et al., 2021). The adoption of such an integrated, multidisciplinary framework represents a critical prerequisite for addressing the multifaceted barriers that have historically hindered the real-world translation of evidence-based non-pharmacological strategies in MASLD management.

**TABLE 3:** Barriers to implementation of non-pharmacological interventions for MASLD.

Level	Barrier category	Specific barriers
Societal	Global society	- Food industry influence promoting ultra-processed foods and added sugars - Increasing sedentary lifestyle - Limited time for physical activity and proper dietary planning - Sociopolitical factors affecting food security and accessibility - Limited public health measures addressing metabolic health - Conflicting and overwhelming nutritional messaging in popular media
Societal	Healthcare system	- Limited reimbursement for lifestyle interventions and nutritional counseling - Paucity of specialized MASLD clinics offering multidisciplinary care - Inadequate incorporation of exercise specialists in liver disease management - Time constraints during clinical consultations limiting lifestyle discussion - Fragmented care between specialties (hepatology, nutrition) - Insufficient screening protocols for early MASLD detection
Societal	Healthcare professionals practice	- Inadequate training of physicians in nutrition science and exercise prescription - Limited knowledge of effective nutritional strategies for MASLD - Insufficient monitoring of adherence to lifestyle modifications - Focus on pharmacological rather than non-pharmacological interventions - Poor interdisciplinary communication between healthcare providers - Limited referral networks to tertiary clinics - Insufficient awareness of current MASLD management guidelines
Societal	Social inequality	- Disparities in access to healthy foods - Socioeconomic barriers to gym memberships and exercise facilities - Limited green spaces in disadvantaged neighborhoods - Higher prevalence of MASLD in marginalized populations with fewer resources - Unequal access to healthcare professionals with MASLD expertise - Financial barriers to obtaining fresh produce and unprocessed foods
Societal	Stigma	- Weight stigma affecting quality of care and health-seeking behaviors - Misconceptions about MASLD - Public perception linking MASLD exclusively to obesity - Healthcare provider bias affecting treatment recommendations
Societal	Education	- Limited public awareness of MASLD as a serious health condition - Poor health literacy regarding metabolic health and liver disease - Inadequate nutritional education in school curricula - Insufficient knowledge about physical activity requirements and benefits - Limited cultural competence in nutrition education materials - Lack of specialized education programs for MASLD management
Individual	Digital environment	- Limited access to evidence-based digital health tools for MASLD management - Poor integration of lifestyle tracking apps with healthcare systems - Digital divide affecting older and disadvantaged populations - Information overload from unverified online sources - Low digital health literacy - Privacy concerns limiting engagement with health monitoring tools
Individual	Social and urban environments	- Limited access to safe areas for physical activity - Food environments dominated by unhealthy options - Workplace environments that discourage physical activity - Urban design promoting car dependency over active transportation - Limited community programs supporting lifestyle change - Inadequate infrastructure for outdoor physical activity
Individual	Cultural environment	- Cultural dietary patterns high in refined carbohydrates and saturated fats - Social gatherings centered around unhealthy eating - Cultural resistance to dietary modifications - Low cultural acceptance of certain exercise modalities - Traditional food practices conflicting with MASLD dietary recommendations - Perception of exercise as leisure rather than medicine
Individual	Health problems	- Fatigue and reduced exercise capacity associated with MASLD - Comorbidities limiting exercise options (joint pain, cardiovascular disease) - Sarcopenia affecting exercise capability - Psychological symptoms (depression, anxiety) affecting motivation - Insulin resistance creating physiological barriers to weight management - Poor sleep quality compromising metabolic health and exercise recovery - Medication side effects interfering with physical activity
Individual	Psychological factors	- Low motivation for sustained lifestyle change - Poor self-efficacy for exercise and dietary adherence - Psychological stress promoting unhealthy eating behaviors - Inadequate coping mechanisms for managing cravings - Emotional eating patterns and food addiction - Limited perceived threat of MASLD complications - Immediate reward preferences over long-term health benefits

**TABLE 4:** Facilitators for implementation of non-pharmacological interventions for MASL**D**.

Level	Enabler category	Specific enabler
Societal	Global society	- Increased recognition of MASLD as a public health priority - Growing awareness of the metabolic health crisis - Development of policies promoting healthy lifestyles - Public health campaigns addressing nutrition literacy and physical activity - Integration of metabolic health measures in preventive healthcare - Food policy initiatives targeting ultra-processed food reduction
Societal	Healthcare system	- Implementation of multidisciplinary MASLD care models - Expanded reimbursement for evidence-based lifestyle interventions - Integration of preventive medicine approaches focusing on metabolic health - Development of standardized MASLD screening protocols - Implementation of 4P medicine (preventive, participatory, personalized, predictive) - Utilization of telemedicine for expanded access to MASLD specialists - Clinical pathways connecting hepatology with lifestyle intervention specialists
Societal	Healthcare education	- Enhanced nutrition and exercise science education in medical curricula - Continuing education programs on non-pharmacological MASLD management - Training in motivational interviewing and health behavior change techniques - Development of specialized MASLD management credentials - Cultural competency training for healthcare providers - Training in interdisciplinary collaboration for metabolic health
Societal	Research and innovation	- Expanded funding for lifestyle intervention research - Development of evidence-based guidelines for exercise in different MASLD stages - Research on personalized nutrition approaches for MASLD phenotypes - Validation of digital health tools for MASLD management - Studies identifying predictors of response to lifestyle interventions - Innovation in accessible exercise equipment and programs
Societal	Awareness and education	- Public education campaigns on MASLD risk factors and management - School-based programs promoting metabolic health literacy - Workplace wellness initiatives addressing nutrition and physical activity - Community-based metabolic health education programs - Media campaigns destigmatizing metabolic conditions - Simplified translation of research findings for public consumption
Individual	Digital technology	- Mobile apps for tracking dietary intake and physical activity - Wearable devices monitoring metabolic health parameters - Telehealth platforms connecting patients with multidisciplinary teams - Virtual coaching for personalized exercise and nutrition guidance - Online communities providing peer support for lifestyle changes - Digital tools for meal planning and nutritional analysis
Individual	Exercise interventions	- Personalized exercise prescriptions based on fitness level and liver health - Combined aerobic and resistance training programs - Supervised exercise sessions with progressive intensity increases - Group-based physical activity promoting social connection - Adapted exercise protocols for patients with comorbidities - Strategies for integrating physical activity into daily routines - Home-based exercise programs requiring minimal equipment
Individual	Nutritional strategies	- Mediterranean dietary pattern emphasizing plant foods and healthy fats - Personalized meal plans addressing individual metabolic profiles - Evidence-based nutritional advice - Strategies for reducing added sugars and refined carbohydrates - Approaches for managing hunger and satiety signals - Techniques for navigating social eating situations - Guidance on reading food labels and identifying ultra-processed foods
Individual	Behavioral support	- Motivational interviewing to address ambivalence about lifestyle change - Goal-setting techniques - Self-monitoring tools for tracking progress and adherence - Cognitive behavioral strategies for managing emotional eating - Stress management techniques supporting metabolic health - Sleep hygiene education and interventions - Relapse prevention strategies for maintaining lifestyle changes
Individual	Social support	- Family-based approaches to dietary modification - Peer support groups for individuals with MASLD - Healthcare provider encouragement and accountability - Workplace wellness programs supporting healthy choices - Community-based physical activity initiatives - Cultural adaptation of lifestyle interventions - Partnerships with community organizations

## 4 Multidisciplinary framework for addressing barriers and leveraging enablers

The conceptual foundation of a multidisciplinary framework for non-pharmacological MASLD management is predicated upon several interdependent core principles–including the strategic integration of diverse professional expertise with clear role delineation; patient-centered interventions that address both societal and individual barriers while leveraging facilitators; early preventive measures to halt disease progression prior to the development of significant fibrosis; tailored approaches responsive to disease severity and comorbidities; optimized monitoring protocols with specific thresholds for intervention adjustment; and judicious incorporation of digital health technologies, accounting for variability in digital literacy (Lara-Romero and Romero-Gómez, 2024). The subsequent analysis examines these fundamental aspects in detail.

### 4.1 Strategic integration of diverse professional expertise

**FIGURE 1:**
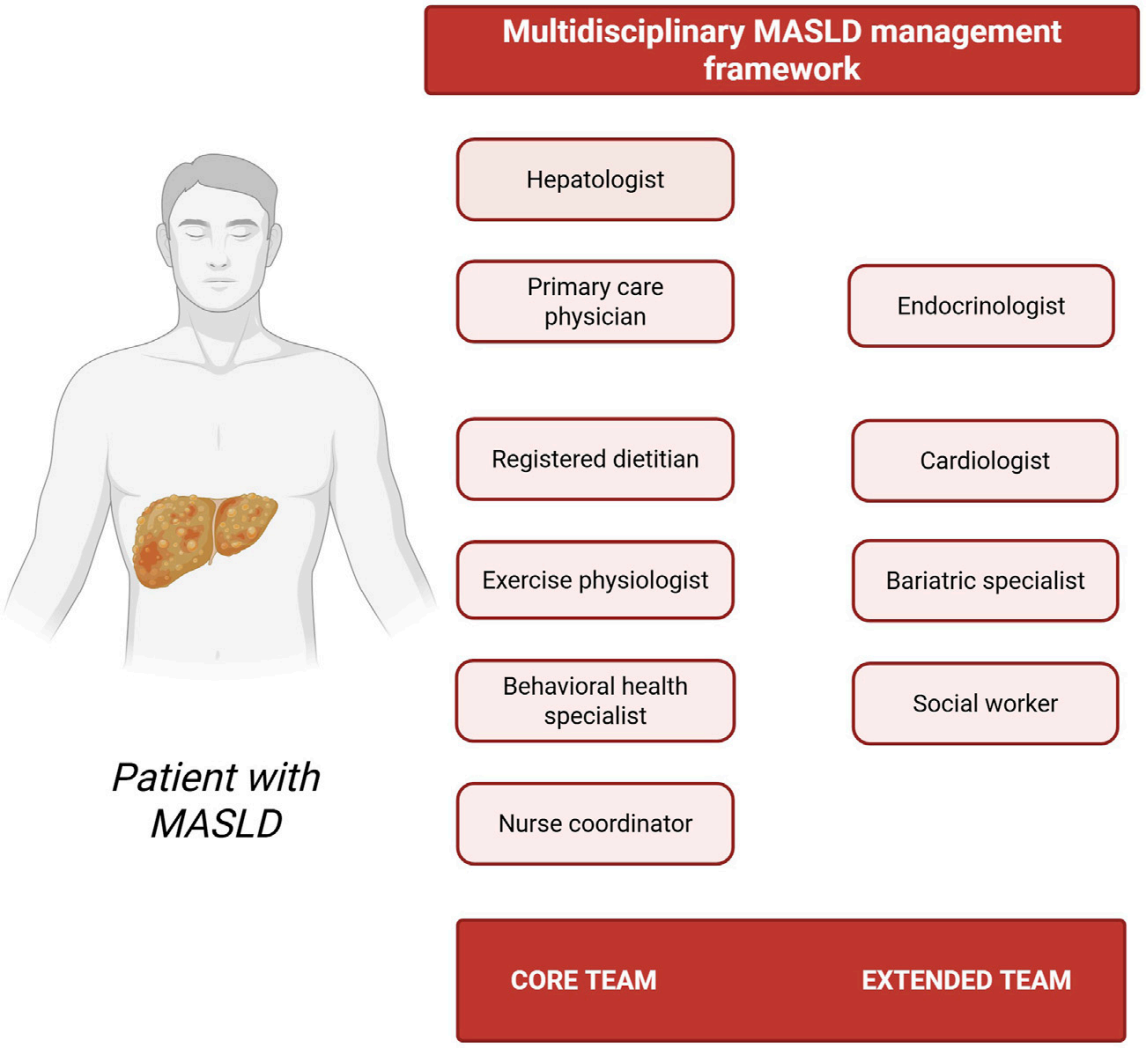
Multidisciplinary MASLD management framework. The schematic representation highlights both core and extended team members, including hepatologist, primary care physician, registered dietitian, exercise physiologist, behavioral health specialist, nurse coordinator, endocrinologist, cardiologist, bariatric specialist, and social worker–emphasizing the importance of coordinated, patient-centered care (Created with BioRender.com).

An optimal multidisciplinary team for non-pharmacological MASLD management should comprise healthcare professionals from various disciplines, each with precisely delineated roles and responsibilities (Figure 1). Specifically, the core team should encompass a hepatologist who performs non-invasive assessment of hepatic steatosis and fibrosis staging, and determines the potential necessity for liver biopsy (Anstee et al., 2022); a primary care physician who orchestrates patient care coordination and ensures longitudinal follow-up (Brandman, 2019); a registered dietitian who designs personalized nutrition plans and delivers practical dietary counseling (Policarpo et al., 2021); an exercise physiologist who formulates individualized physical activity prescriptions (Haus et al., 2013); a behavioral health specialist who addresses psychological barriers to treatment adherence (Balakrishnan et al., 2023); and a nurse coordinator who facilitates patient education and monitors intervention adherence (Vacca, 2020). Extended team members may also be incorporated as dictated by specific patient requirements. These specialists can include an endocrinologist for management of diabetes and related metabolic disorders (Lomonaco et al., 2011); a cardiologist for comprehensive cardiovascular risk assessment (Sîrbu et al., 2016); a bariatric specialist for advanced weight management interventions in patients with morbid obesity (Głuszyńska et al., 2021); and a social worker to address socioeconomic determinants of health (Ye et al., 2024). This comprehensive interprofessional structure may ultimately ensure that all dimensions of MASLD management are addressed through the synchronized efforts of specialized experts operating within a cohesive framework.

### 4.2 Patient-centered interventions

For individuals with MASLD, patient-centered care necessitates understanding the complex interplay of barriers and facilitators that influence adherence and outcomes to non-pharmacological management (Eskridge et al., 2023). Unfortunately, healthcare system barriers significantly impact patient-centered care delivery–including limited reimbursement for lifestyle interventions (Gobble et al., 2022; Freeman et al., 2021), fragmented care between specialties (Brahmania et al., 2024), and insufficient screening protocols (Ilagan-Ying et al., 2023). Potential solutions may include developing cost-effectiveness data supporting lifestyle interventions (Schulz et al., 2014), implementing shared electronic health records (Li et al., 2022), and developing standardized screening pathways that include comprehensive metabolic and liver health assessments (Arora et al., 2024). Professional practice barriers such as inadequate training in nutrition and exercise prescription (Mogre et al., 2018), excessive focus on pharmacological rather than behavioral interventions (Hossain et al., 2016), and insufficient monitoring of adherence (Zeng et al., 2024) also require targeted solutions. These challenges can be overcome through continuing education programs (Donghia et al., 2025), established referral pathways (Chan et al., 2023), and digital tracking tools integrated with electronic health records (Kirilov, 2024). Importantly, the provision of effective patient-centered care for MASLD necessitates the systematic identification and targeted remediation of individual-level barriers. Health-related hurdles affecting exercise capability–including fatigue, depression, sleep disorders (Mostafa et al., 2024), reduced exercise capacity (Deshpande et al., 2024), and sarcopenia (Li et al., 2024) – necessitate tailored adaptations. These can be addressed through low-intensity, short-duration activities with gradual progression, personalized exercise prescriptions accommodating joint pain and functional limitations, and incorporation of resistance training with appropriate progression protocols. Psychological barriers–including low motivation for lifestyle change and poor self-efficacy for exercise and diet adherence–can be mitigated through motivational interviewing techniques, setting small achievable goals, and providing guided experiences with supportive feedback (Hardcastle et al., 2015). Another aspect that should be carefully considered is that patient-centered approaches must account for social and cultural contexts. As previously discussed, limited public awareness of MASLD (Kopka et al., 2024), poor health literacy (Donghia et al., 2025), and challenging food environments (Paik et al., 2024) represent significant barriers. Addressing these aspects may require public education campaigns highlighting the reversibility of MASLD through lifestyle changes, development of culturally appropriate educational materials, and strategic partnerships with community organizations to improve healthy food access (Allen et al., 2025). On a separate note, environmental constraints such as limited access to exercise facilities (Hillsdon et al., 2007) and food environments promoting unhealthy choices (Paik et al., 2024) necessitate creative solutions. Home-based exercise programs requiring minimal equipment, identification of safe walking areas, and guidance for meal planning and preparation strategies that incorporate cultural food preferences can potentially address these challenges. Additionally, social support enablers–including family-based approaches to dietary modification, peer support groups for individuals with MASLD, and community partnerships–can significantly enhance patient-centered care. Effective implementation may involve engaging family members in nutrition education sessions and establishing both in-person and virtual peer support groups to broaden the spectrum of available resources.

### 4.3 Early preventive measures

Early detection of MASLD during pre-fibrotic stages permits capitalizing on preserved hepatic regenerative potential before the establishment of architectural changes such as fibrous septa and regenerative nodule formation (Albert and Wood, 2024). Evidence-based nutritional strategies represent a fundamental cornerstone of preventive anti-fibrotic strategies (Gao et al., 2023). Specifically, Mediterranean dietary patterns (Miryan et al., 2023), personalized nutritional approaches (Marin-Alejandre et al., 2021), as well as a high intake of vegetables, fruits, vegetable oils, low-fat dairy products, white meat, and nuts (Soleimani et al., 2019) may confer substantial preventive benefits when implemented during the initial stages of the disease trajectory. To optimize efficacy, these nutritional interventions should be ideally tailored to individual preferences and operationalized through practical meal plans that incorporate cultural considerations and sustainable dietary modifications. In parallel, early implementation of structured physical activity programs may facilitate the establishment of sustainable habits prior to disease progression that might otherwise limit exercise capacity (Chun et al., 2023). The utilization of validated rating of perceived exertion scales ensures appropriate exercise intensity, while individualized modifications accommodate patients with pre-existing functional limitations (Glass et al., 2022). We believe that comprehensive early preventive approaches must systematically address modifiable risk factors–including excessive alcohol consumption, suboptimally controlled diabetes mellitus, dyslipidemia, and obesity (Xie et al., 2023). Proactive management of these factors can mitigate the cumulative effects of multiple metabolic derangements before significant hepatocellular damage manifests. In this scenario, prevention-focused educational interventions should be strategically directed toward both the general population and high-risk clinical cohorts (Schattenberg et al., 2023). These endeavors should be coupled with culturally appropriate informational resources and robust community partnerships to optimize their preventive efficacy and population-level impact.

### 4.4 Tailored approaches responsive to disease severity and comorbidities

The optimal implementation of non-pharmacological strategies in MASLD management necessitates systematic adaptation of therapeutic modalities based on a comprehensive assessment of disease severity, fibrosis stage, and comorbid conditions. This personalized therapeutic paradigm ensures that interventions specifically target the underlying pathophysiological mechanisms while accommodating the limitations imposed by coexisting conditions (Lonardo et al., 2021). For patients exhibiting simple steatosis without significant inflammatory activity or fibrosis, the therapeutic emphasis should remain on lifestyle modifications coupled with vigilant monitoring protocols (Lonardo et al., 2021; Valenzuela-Vallejo et al., 2023). As disease severity progresses, more intensive interventions become imperative, including structured dietary regimens, supervised exercise protocols, and judicious consideration of pharmacological adjuncts when clinically indicated (Valenzuela-Vallejo et al., 2023). Notably, MASLD frequently manifests within a constellation of metabolic comorbidities requiring coordinated management strategies (Targher et al., 2024). In this context, the previously described multidisciplinary care team must meticulously adapt interventions to address concurrent conditions including diabetes mellitus, cardiovascular disease, dyslipidemia, and visceral adiposity, ensuring therapeutic approaches maintain coherence rather than introducing contradictory elements. This necessitates specific vigilance regarding potential pharmacological interactions, contraindications to specific exercise modalities, and nutritional considerations that should simultaneously address multiple metabolic derangements. The presence and extent of hepatic fibrosis necessitates specific modifications to non-pharmacological interventions (Semmler et al., 2021). For patients with advanced fibrosis or established cirrhosis, nutritional interventions must address protein requirements that differ between compensated and decompensated liver disease, sodium restrictions in the presence of ascites, and micronutrient deficiencies characteristic of advanced cirrhosis (Mendez-Guerrero et al., 2024). Therapeutic adaptations must also account for physical and functional limitations that may impact intervention adherence (Glass et al., 2022). For patients with sarcopenia, mobility restrictions, or cardiovascular disease, exercise prescriptions should incorporate modified modalities such as seated exercises, aquatic therapy, or adapted movements that preserve therapeutic efficacy while minimizing risk. These adaptations should follow a progressive implementation model, commencing with supervised sessions that gradually transition to modified home-based programs with appropriate safety parameters. Additionally, the complexity of non-pharmacological interventions must be calibrated against the patient’s capacity to implement recommendations. For individuals with multiple comorbidities already managing complex treatment regimens, therapeutic approaches may require strategic prioritization and phased implementation to prevent exceeding adherence capacity. This strategic approach may ultimately ensure that interventions remain feasible while maximizing their potential benefit across the disease spectrum and comorbidity profile.

### 4.5 Optimized monitoring protocols

**FIGURE 2:**
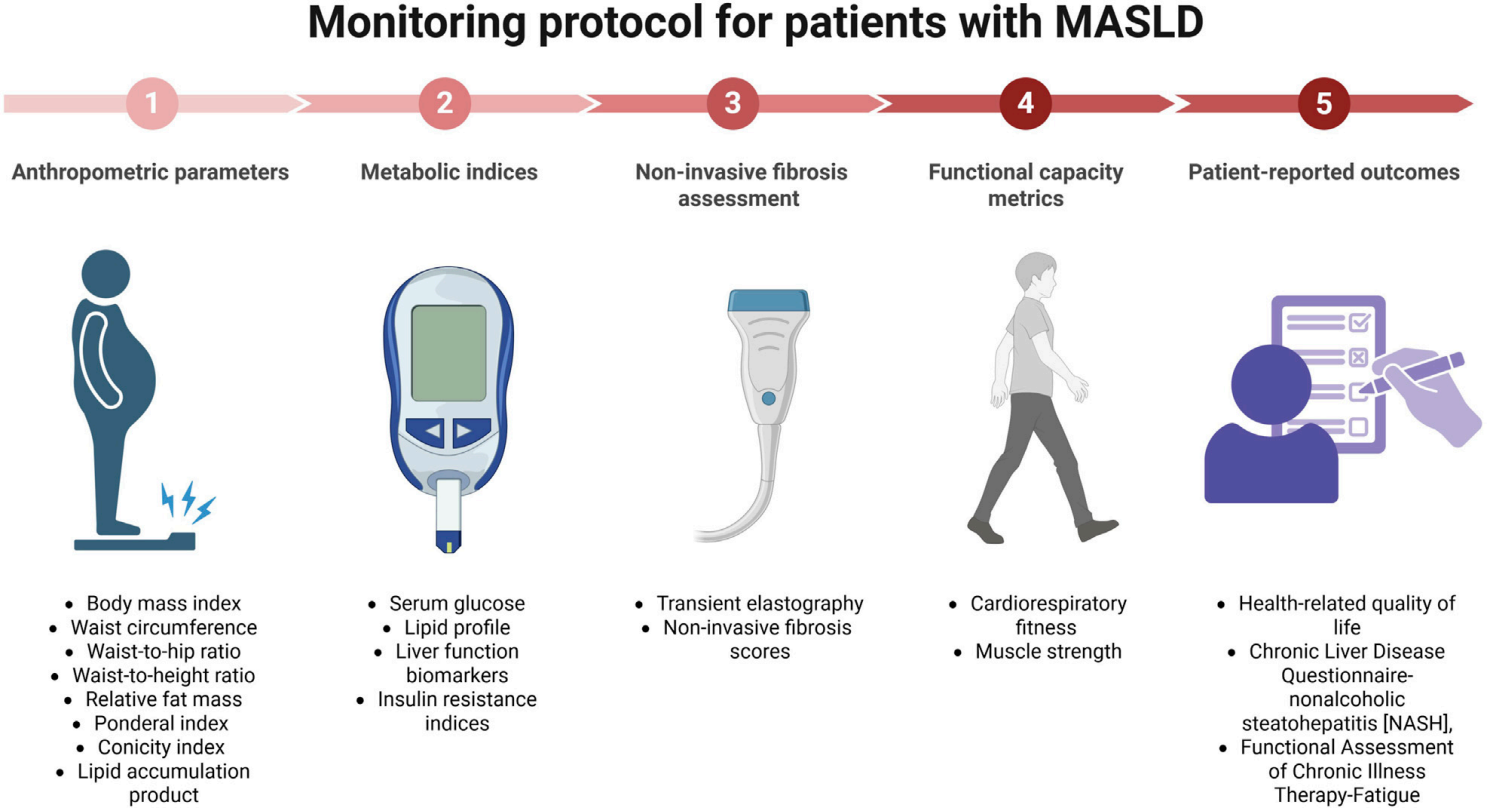
Comprehensive monitoring protocol for patients with MASLD. Overview of a structured monitoring protocol for patients with MASLD, detailing five key domains: (1) anthropometric parameters (e.g., body mass index, waist circumference, fat mass indices), (2) metabolic indices (e.g., serum glucose, lipid profile, liver function biomarkers, insulin resistance), (3) non-invasive fibrosis assessment (e.g., transient elastography, fibrosis scores), (4) functional capacity metrics (e.g., cardiorespiratory fitness, muscle strength), and (5) patient-reported outcomes (e.g., health-related quality of life, disease-specific questionnaires) (Created with BioRender.com).

An optimized management of MASLD through non-pharmacological interventions necessitates the implementation of comprehensive monitoring frameworks that systematically evaluate intervention adherence, physiological adaptations, and biomarkers of disease progression (Noureddin et al., 2024). Such protocols should incorporate evidence-derived thresholds for therapeutic modifications while retaining sufficient flexibility to accommodate heterogeneity in individual treatment responses. Rigorous monitoring frameworks may encompass multiple assessment domains–including anthropometric parameters, metabolic indices, non-invasive fibrosis assessment tools, functional capacity metrics, and patient-reported outcomes (Figure 2). This multidimensional approach ensures comprehensive evaluation of both disease-specific parameters and global health indices. Appropriate assessment intervals that optimize the balance between timely intervention modifications and minimization of resource utilization and patient burden are also advisable. Initial comprehensive assessments should establish baseline values across all relevant parameters, followed by more frequent evaluations during the initial intervention phase to capture rapid modifications in modifiable factors. As clinical stability is achieved, monitoring intervals may be judiciously extended while maintaining vigilance for indicators of disease progression or suboptimal response. The monitoring framework should also incorporate systematic assessment of intervention adherence across nutritional, physical activity, and behavioral domains. Upon identification of adherence barriers (Castera et al., 2025), the monitoring protocol should trigger specific supportive interventions including therapeutic goal recalibration, systematic barrier analysis, and provision of supplementary resources or educational interventions. Importantly, contemporary monitoring frameworks must incorporate systematic assessment of patient-reported outcomes including treatment satisfaction indices and health-related quality of life metrics (Younossi et al., 2022). These subjective measures provide essential context for the interpretation of physiological parameters and guide therapeutic adjustments to ensure interventions remain congruent with patient priorities and capabilities (Barberá et al., 2024). Regular assessment of these outcomes may ultimately facilitate collaborative decision-making regarding management modifications and supports sustained patient engagement throughout the therapeutic continuum.

### 4.6 Judicious incorporation of digital health technologies

Digital health technologies offer significant potential to improve non-pharmacological MASLD management through enhanced monitoring capabilities, expanded access to specialized care, and delivery of personalized interventions (Allen et al., 2024). As previously discussed, digital technologies may serve as significant enablers through mobile health applications for dietary intake documentation and physical activity quantification, telehealth platforms facilitating patient-specialist connectivity, and wearable biosensors monitoring physiological and behavioral parameters (Buzcu et al., 2024; Albers et al., 2023; Keshet et al., 2023). These technological modalities can extend the therapeutic reach of healthcare providers beyond traditional clinical environments, enabling continuous monitoring and supportive interventions between scheduled clinical encounters. For MASLD management specifically, digital tools may facilitate precise dietary assessment (Zuppinger et al., 2022) and objective physical activity quantification (Kamel Boulos and Yang, 2021). Nonetheless, the reliance on technological solutions must acknowledge and systematically address variations in digital literacy across diverse patient populations. In this setting, effective implementation approaches should include structured orientation protocols for recommended applications, establishment of tiered support systems for users with limited technological proficiency, development of standardized protocols for virtual consultations, and incorporation of progressive technology adoption strategies calibrated to individual capability and comfort. For patients with limited digital literacy, hybrid implementation models combining traditional intervention delivery with incremental technological integration may optimize engagement while progressively building technological self-efficacy. The selection of technological interventions should be guided by robust evidence regarding their clinical effectiveness, user interface accessibility, and appropriateness for specific patient populations. Additionally, implementation strategies should prioritize technologies with demonstrated efficacy in MASLD management (Kaewdech et al., 2024; Alkhouri et al., 2023) while considering multifaceted factors including cost implications, accessibility across socioeconomic strata, data security protocols, and interoperability capabilities with existing healthcare information systems. This evidence-based selection process helps ensure that technological interventions deliver measurable clinical benefits rather than merely increasing system complexity without corresponding improvements in patient outcomes. While technological solutions offer considerable advantages, we believe that they must complement rather than supplant interpersonal connections in healthcare delivery. Finally, the implementation of novel technological solutions–including artificial intelligence–necessitates careful consideration of ethical dimensions including data privacy protections, informed consent processes, and potential exacerbation of existing healthcare disparities (Pugliese et al., 2025). These ethical considerations should be systematically addressed through structured clinical educational frameworks that equip healthcare professionals with both technological competencies and ethical awareness in digital health implementation.

## 5 Discussion

This qualitative synthesis of the available evidence has provided a comprehensive elucidation of barriers and facilitators influencing the real-world implementation of non-pharmacological strategies for MASLD management. Our findings suggest that, to optimize effectiveness in real-world settings, nutritional and physical activity interventions require coordinated, multilevel strategies that concurrently address both societal and individual barriers, while strategically harnessing facilitating factors. In response, the multidisciplinary framework presented herein outlines a systematic implementation approach encompassing the full spectrum from primary prevention to the management of advanced hepatic pathology. This framework emphasizes the critical need for therapeutic customization based on disease severity, individual patient contexts, and sociocultural determinants. Moreover, it highlights the essential shift from fragmented care models toward integrated, patient-centered paradigms, wherein hepatologists, primary care providers, nutritionists, exercise physiologists, and behavioral health specialists operate collaboratively with harmonized objectives and well-defined communication pathways.

Several critical insights emerge from the reviewed evidence. Foremost among these is the recognition that early intervention is of paramount importance. Although growing evidence indicates that even cirrhosis may be reversible under certain conditions (Jung and Yim, 2017; Sohrabpour et al., 2012), initiating dietary and physical activity interventions prior to the development of significant fibrosis in patients with MASLD represents a more rational and effective strategy than attempting to reverse advanced liver pathology. Early intervention may also benefit from a lower burden of MASLD-associated comorbidities, such as fatigue and sarcopenia, which are known to impede successful lifestyle modifications. However, barriers to implementation extend beyond the individual patient level, permeating healthcare systems, professional education curricula, and broader societal structures. These observations underscore the necessity for coordinated, multi-tiered interventions. In this context, digital health technologies can offer both opportunities and challenges, presenting a complex landscape that demands meticulous implementation strategies. It is also noteworthy that cultural determinants and psychological factors might exert a profound influence on adherence to lifestyle modifications. This highlights the need for personalized, culturally sensitive approaches, rather than reliance on standardized recommendations, to enhance the effectiveness and sustainability of interventions.

The operational agenda delineated herein addresses critical knowledge gaps, particularly concerning personalized intervention strategies and implementation methodologies across diverse healthcare environments. Future research should prioritize the development and rigorous validation of cost-effective, scalable delivery models for evidence-based lifestyle interventions that can be systematically integrated into routine clinical practice within heterogeneous healthcare systems. Health policymakers and healthcare leadership should recognize that meaningful advancements in MASLD management require substantial investment in preventive strategies and lifestyle intervention infrastructure. This includes expanding reimbursement frameworks for evidence-based lifestyle programs, systematically incorporating nutrition specialists and exercise physiologists into hepatology care teams, enhancing professional education in nutritional science and physical activity prescription, and implementing public health initiatives that improve food environment quality and opportunities for physical activity. For clinicians, the current evidence synthesis offers pragmatic guidance for the implementation of multidisciplinary care models and for systematically overcoming common barriers to sustainable lifestyle modification. The proposed framework underscores that, although weight reduction remains a key therapeutic objective, an exclusive focus on anthropometric outcomes may be counterproductive. Instead, prioritizing improvements in metabolic health through sustainable dietary patterns and structured physical activity regimens represents a more constructive and patient-centered approach for many individuals with MASLD.

However, our findings need to be interpreted in the context of several limitations. First, we recognize that socioeconomic determinants may play a fundamental role in shaping adherence to non-pharmacological interventions for MASLD, as they influence access to resources, health literacy, and the ability to sustain lifestyle changes. Factors such as income disparities, limited access to healthy foods, and financial barriers to exercise facilities can exacerbate disease progression, particularly in marginalized populations. To enhance adherence, our proposed framework integrates targeted strategies like including social workers in multidisciplinary teams to address these determinants directly, alongside community partnerships and policy advocacy for equitable resource distribution. While we acknowledge that stratifying barriers and facilitators by specific socioeconomic factors would provide further insight into intervention tailoring, this level of granularity was not feasible within the scope of our current synthesis. Future research should prioritize detailed stratification to better understand how socioeconomic subgroups experience distinct challenges and to develop even more targeted approaches. Second, the effectiveness of any management framework depends on its adaptation to specific regional and cultural contexts. As each community presents a unique combination of barriers and facilitators, a one-size-fits-all approach to non-pharmacological interventions cannot be considered satisfactory. In the future, it will be essential to conduct regional studies and culturally adapt research to address the distinct socioeconomic conditions, healthcare access, and social determinants of health that characterize different populations. Third, when implementing nutritional interventions–particularly those based on the Mediterranean dietary pattern–it is crucial to emphasize to both patients and healthcare providers that the primary goal is to select foods with comparable nutritional profiles, rather than sourcing items exclusively from Mediterranean countries. This includes an emphasis on plant-based foods and minimally processed ingredients. To enhance feasibility, sustainability, and adherence, the dietary approaches should be adapted to local food availability and culinary traditions. Furthermore, recommendations to increase fruit intake should be accompanied by careful guidance. Although fruits are excellent sources of vitamins, fiber, and beneficial phytochemicals, some varieties are also high in fructose, which–when consumed in excess–may contribute to hepatic fat accumulation and disease progression. Clinical advice should therefore emphasize moderation, encourage the selection of whole, fiber-rich fruits over fruit juices or sweetened fruit products, and be individualized to consider each patient’s metabolic and clinical profile. An additional limitation of this synthesis is that the barriers and facilitators identified were not stratified according to disease duration. It is likely that patients with longstanding MASLD/MASH may experience distinct challenges–such as intervention fatigue, cumulative comorbidities, or adaptive behaviors–compared to those who are recently diagnosed, whose primary barriers may relate more to health literacy or initial motivation. Future research should specifically address how barriers and facilitators to non-pharmacological management change over the disease trajectory, to inform the design of stage-specific interventions that support sustained adherence. We also acknowledge that the evidence base synthesized in this review is derived from studies exhibiting substantial heterogeneity in design, population characteristics, intervention modalities, and outcome measures. This variability may constrain the direct comparability of findings and limit the strength of our synthesized conclusions. As with all qualitative syntheses, the potential for selection and interpretation bias during coding, theme development, and synthesis remains an inherent limitation. Furthermore, the transferability of these results to health systems and countries with different MASLD burdens may be restricted. Future research should aim to validate these identified barriers, facilitators, and proposed frameworks in diverse international contexts, especially in regions facing a high or increasing prevalence of MASLD.

In conclusion, while non-pharmacological strategies are widely acknowledged as foundational in MASLD management, their full therapeutic potential remains unrealized without systematic operationalization that addresses implementation barriers and leverages facilitators. This necessitates progressing beyond broad conceptual acceptance toward the adoption of evidence-based frameworks that afford lifestyle interventions methodological rigor comparable to pharmacotherapies–including standardized protocols, longitudinal outcome monitoring, and integration into clinical care pathways. Future efforts should concentrate on bridging persistent implementation gaps through precision nutrition strategies, culturally adapted exercise prescriptions, and digital health solutions that enhance scalability. Concurrently, advancing biomarker-driven monitoring frameworks and interdisciplinary training programs will strengthen the evidence-based application of these interventions. Collectively, these measures will enable the hepatology community to transition from viewing lifestyle interventions as theoretically foundational to delivering them as practically transformative, thereby substantially reducing the global burden of MASLD-related morbidity.
